# 1-(4-Chlorophenyl)-3-(3-chloro­pro­pionyl)thio­urea

**DOI:** 10.1107/S1600536813028511

**Published:** 2013-10-23

**Authors:** Bohari M. Yamin, Siti K. C. Soh, Siti Fairus M. Yusoff

**Affiliations:** a School of Chemical Sciences and Food Technology, Universiti Kebangsaan Malaysia, 43600 Bangi, Selangor, Malaysia

## Abstract

In the title compound, C_10_H_10_Cl_2_N_2_OS, the mol­ecule adopts a *trans–cis* conformation with respect to the position of the carbonyl group and the chloro­phenyl groups relative to the thiono group across the C—N bonds. The mol­ecule is stabilized by an N—H⋯O hydrogen bond. In the crystal, mol­ecules are linked by N—H⋯S and C—H⋯O hydrogen bonds, forming zigzag chains along the *b*-axis direction. C—H⋯π inter­actions are also present.

## Related literature

For bond-length data, see: Allen *et al.* (1987 ▶). For related thio­urea derivatives, see: Othman *et al.* (2010 ▶); Yamin *et al.* (2011 ▶); Yamin & Othman (2011 ▶); Yusof *et al.* (2011 ▶).
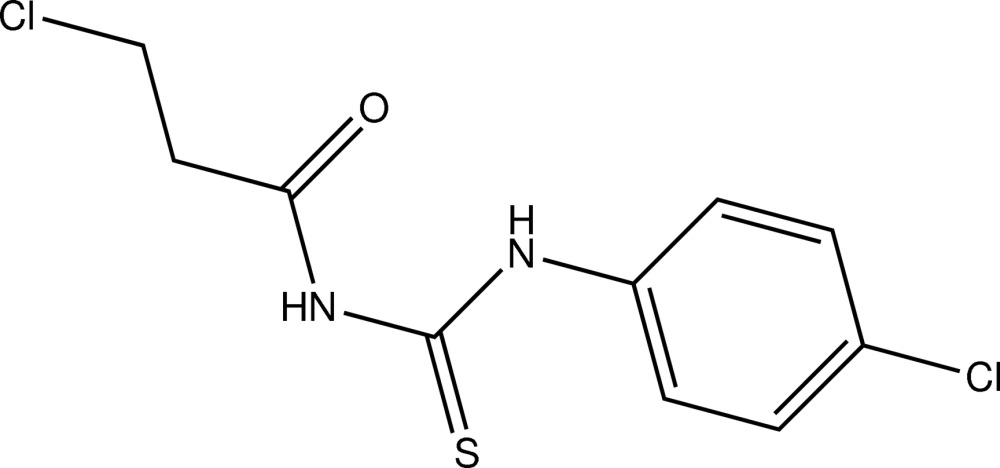


## Experimental

### 

#### Crystal data

C_10_H_10_Cl_2_N_2_OS
*M* *_r_* = 277.16
Triclinic, 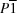

*a* = 5.5151 (16) Å
*b* = 9.045 (3) Å
*c* = 12.387 (4) Å
α = 101.000 (5)°
β = 94.027 (5)°
γ = 94.780 (5)°
*V* = 602.1 (3) Å^3^
*Z* = 2
Mo *K*α radiation
μ = 0.69 mm^−1^
*T* = 298 K
0.47 × 0.21 × 0.08 mm

#### Data collection

Bruker SMART APEX CCD area-detector diffractometer
Absorption correction: multi-scan (*SADABS*; Bruker, 2000 ▶) *T*_min_ = 0.737, *T*_max_ = 0.947
5947 measured reflections
2227 independent reflections
1698 reflections with *I* > 2σ(*I*)
*R*_int_ = 0.035

#### Refinement

*R*[*F* ^2^ > 2σ(*F* ^2^)] = 0.053
*wR*(*F* ^2^) = 0.116
*S* = 1.22
2227 reflections
145 parameters
H-atom parameters constrained
Δρ_max_ = 0.25 e Å^−3^
Δρ_min_ = −0.29 e Å^−3^

### 

Data collection: *SMART* (Bruker, 2000 ▶); cell refinement: *SAINT* (Bruker, 2000 ▶); data reduction: *SAINT*; program(s) used to solve structure: *SHELXTL* (Sheldrick, 2008 ▶); program(s) used to refine structure: *SHELXTL*; molecular graphics: *SHELXTL*; software used to prepare material for publication: *SHELXTL*, *PARST* (Nardelli, 1995 ▶) and *PLATON* (Spek, 2009 ▶).

## Supplementary Material

Crystal structure: contains datablock(s) global, I. DOI: 10.1107/S1600536813028511/lr2115sup1.cif

Structure factors: contains datablock(s) I. DOI: 10.1107/S1600536813028511/lr2115Isup2.hkl

Supplementary material file. DOI: 10.1107/S1600536813028511/lr2115Isup3.cml

Additional supplementary materials:  crystallographic information; 3D view; checkCIF report

## Figures and Tables

**Table 1:** Hydrogen-bond geometry (Å, °) *Cg*1 is the centroid of the C5–C10 benzene ring.

*D*—H⋯*A*	*D*—H	H⋯*A*	*D*⋯*A*	*D*—H⋯*A*
N2—H2*A*⋯O1	0.86	1.92	2.646 (4)	141
N1—H1*A*⋯S1^i^	0.86	2.52	3.367 (3)	169
C9—H9*A*⋯O1^ii^	0.93	2.55	3.402 (5)	152
C1—H1*B*⋯*Cg*1^iii^	0.97	2.92	3.690 (4)	137

Symmetry codes: (i) 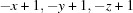; (ii) 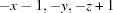; (iii) 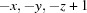.

**Fig. 1.**
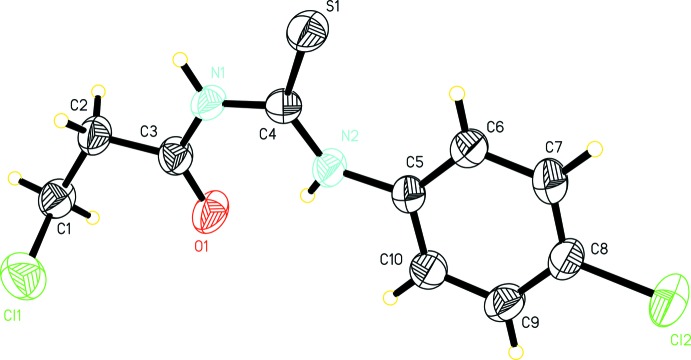
Molecular structure of (I) with 50% probability displacement ellipsoids

**Fig. 2.**
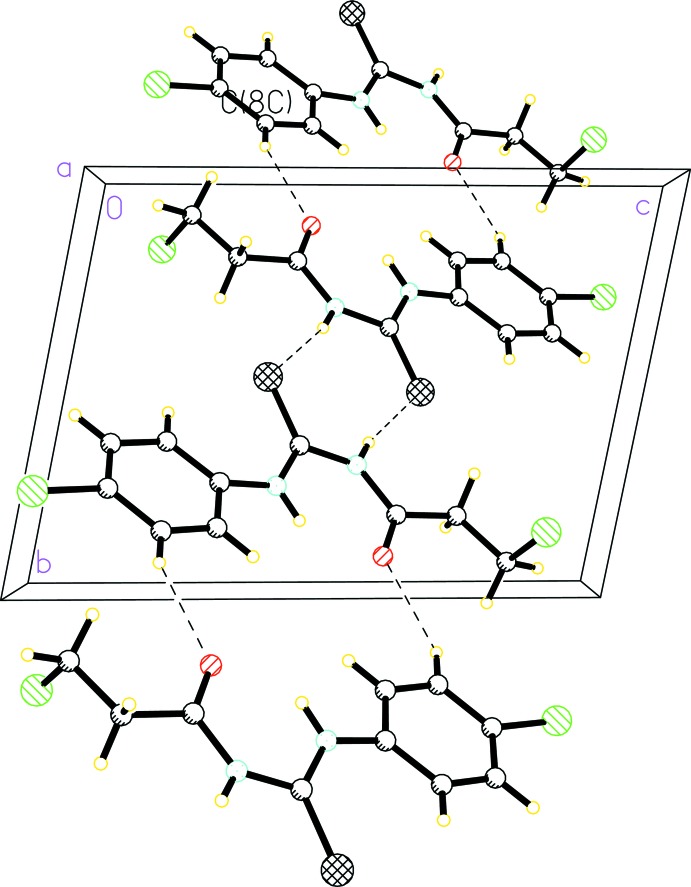
Molecular packing of (I) in the unit cell viewed down the a axis

## supplementary crystallographic information

### 1. Comment

There are not many halogenocarbonyl reported compare to other aroyl or alkoyl-thioureas. N-(4-chlorobutanoyl)-N'-phenylthiourea (Yamin *et al.*, 2011), N-(4-chlorobutanoyl)-N'-(2-fluorophenyl)thiourea (Yusof *et al.*, 2011) and N-(4-bromobutanoyl)-N'-phenylthiourea (Yamin & Othman, 2011) are some examples of halogenobutanoyl thiourea. The title compound is a 3-chloropropionyl thiourea similar to N-(3-chloropropionyl)-N'- phenylthiourea (Othman *et al.* 2010) except the presence of chlorine atom at the para-position of the phenyl ring.

The whole molecule is not planar (Fig. 1) because of the dihedral angle of 14.36 (12)° between chlorophenylamine, Cl2/(C5-C10)/N2, and thiourea C5/N2/C4/N1/S1 fragments. Both fragments are each planar with maximum deviation of 0.015 (3)Å for N2 atom from the least square plane of the thiourea fragment. The bond lengths and angles are in normal ranges (Allen *et al.* 1987). The molecule maintains trans-cis configuration with respect to the position of chloropropionyl and chlorophenyl against the thiono group about N1-C4 and N2-C4 bonds, respectively.

There is an intramolecular N2-H2A···O1 hydrogen bonds . In the crystal packing, the molecules are linked by N1-H1A···S1 and C9-H9A···O1 intermolecular hydrogen bonds (symmetry codes as in Table 1) to form zigzag linear chains extended along b axis (Fig. 2). In addition, there is also a C1-H1B···π bond with the centroid benzene ring Cg1, (C5-C10) (Table 2).

### 2. Experimental

4-chloroaniline (1.27 g, 0.01 mol) disolved in 30 ml of acetone was added into a solution of 3-chloropropionyl isothiocyanate (1.49 g, 0.01 mol) in 30 ml acetone. The mixture was refluxed for 2 hours. The solution was filtered and left to evaporate at room temperature. The white precipitate obtained after a few days, was washed with water and cold ethanol. The colorless crystals were obtained by recrystallization from ethanol.

### 3. Refinement

After location in the difference map, the H-atoms attached to the C and N atoms were fixed geometrically at ideal positions and allowed to ride on the parent atoms with C—H = 0.93-0.97 Å, N—H = 0.86 Å and with *U*_iso_(H)=1.2*U*_eq_(C or N).

### Crystal data

**Table:**

C_10_H_10_Cl_2_N_2_OS	*V* = 602.1 (3) Å^3^
*M**_r_* = 277.16	*Z* = 2
Triclinic, *P*1	*F*(000) = 284
Hall symbol: -P 1	*D*_x_ = 1.529 Mg m^−^^3^
*a* = 5.5151 (16) Å	Mo *K*α radiation, λ = 0.71073 Å
*b* = 9.045 (3) Å	µ = 0.69 mm^−^^1^
*c* = 12.387 (4) Å	*T* = 298 K
α = 101.000 (5)°	Block, colourless
β = 94.027 (5)°	0.47 × 0.21 × 0.08 mm
γ = 94.780 (5)°	

### Data collection

**Table:**

Bruker SMART APEX CCD area-detector diffractometer	2227 independent reflections
Radiation source: fine-focus sealed tube	1698 reflections with *I* > 2σ(*I*)
Graphite monochromator	*R*_int_ = 0.035
Detector resolution: 83.66 pixels mm^-1^	θ_max_ = 25.5°, θ_min_ = 1.7°
ω scans	*h* = −6→6
Absorption correction: multi-scan (*SADABS*; Bruker, 2000)	*k* = −10→10
*T*_min_ = 0.737, *T*_max_ = 0.947	*l* = −14→14
5947 measured reflections	

### Refinement

**Table:**

Refinement on *F*^2^	Primary atom site location: structure-invariant direct methods
Least-squares matrix: full	Secondary atom site location: difference Fourier map
*R*[*F*^2^ > 2σ(*F*^2^)] = 0.053	Hydrogen site location: inferred from neighbouring sites
*wR*(*F*^2^) = 0.116	H-atom parameters constrained
*S* = 1.22	*w* = 1/[σ^2^(*F*_o_^2^) + (0.0289*P*)^2^ + 0.4821*P*] where *P* = (*F*_o_^2^ + 2*F*_c_^2^)/3
2227 reflections	(Δ/σ)_max_ < 0.001
145 parameters	Δρ_max_ = 0.25 e Å^−^^3^
0 restraints	Δρ_min_ = −0.29 e Å^−^^3^

### Special details

**Table:**

Geometry. All esds (except the esd in the dihedral angle between two l.s. planes) are estimated using the full covariance matrix. The cell esds are taken into account individually in the estimation of esds in distances, angles and torsion angles; correlations between esds in cell parameters are only used when they are defined by crystal symmetry. An approximate (isotropic) treatment of cell esds is used for estimating esds involving l.s. planes.
Refinement. Refinement of F^2^ against ALL reflections. The weighted R-factor wR and goodness of fit S are based on F^2^, conventional R-factors R are based on F, with F set to zero for negative F^2^. The threshold expression of F^2^ > 2sigma(F^2^) is used only for calculating R-factors(gt) etc. and is not relevant to the choice of reflections for refinement. R-factors based on F^2^ are statistically about twice as large as those based on F, and R- factors based on ALL data will be even larger.

### Fractional atomic coordinates and isotropic or equivalent isotropic displacement parameters (Å^2^)

**Table:**

	*x*	*y*	*z*	*U*_iso_*/*U*_eq_	
Cl1	−0.1359 (2)	0.15827 (13)	0.11875 (8)	0.0690 (3)	
Cl2	−0.63977 (19)	0.26746 (13)	0.95531 (9)	0.0666 (3)	
S1	0.27725 (18)	0.51842 (10)	0.63440 (8)	0.0481 (3)	
O1	−0.0306 (5)	0.1007 (3)	0.3804 (2)	0.0569 (7)	
N1	0.2306 (5)	0.3097 (3)	0.4537 (2)	0.0405 (7)	
H1A	0.3596	0.3612	0.4406	0.049*	
N2	−0.0333 (5)	0.2657 (3)	0.5811 (2)	0.0422 (7)	
H2A	−0.0743	0.1856	0.5312	0.051*	
C1	0.1271 (7)	0.0805 (4)	0.1674 (3)	0.0491 (9)	
H1B	0.0777	−0.0151	0.1878	0.059*	
H1C	0.2346	0.0610	0.1087	0.059*	
C2	0.2629 (7)	0.1865 (4)	0.2656 (3)	0.0465 (9)	
H2B	0.4247	0.1547	0.2774	0.056*	
H2C	0.2824	0.2874	0.2494	0.056*	
C3	0.1378 (6)	0.1931 (4)	0.3701 (3)	0.0419 (8)	
C4	0.1477 (6)	0.3573 (4)	0.5567 (3)	0.0369 (8)	
C5	−0.1697 (6)	0.2764 (4)	0.6739 (3)	0.0382 (8)	
C6	−0.1040 (7)	0.3699 (4)	0.7750 (3)	0.0503 (10)	
H6A	0.0389	0.4351	0.7854	0.060*	
C7	−0.2499 (7)	0.3672 (4)	0.8611 (3)	0.0522 (10)	
H7A	−0.2060	0.4310	0.9291	0.063*	
C8	−0.4593 (6)	0.2700 (4)	0.8458 (3)	0.0436 (9)	
C9	−0.5279 (6)	0.1755 (4)	0.7458 (3)	0.0467 (9)	
H9A	−0.6708	0.1104	0.7358	0.056*	
C10	−0.3824 (6)	0.1788 (4)	0.6610 (3)	0.0442 (9)	
H10A	−0.4269	0.1143	0.5932	0.053*	

### Atomic displacement parameters (Å^2^)

**Table:**

	*U*^11^	*U*^22^	*U*^33^	*U*^12^	*U*^13^	*U*^23^
Cl1	0.0701 (7)	0.0818 (8)	0.0521 (6)	0.0101 (6)	0.0018 (5)	0.0059 (5)
Cl2	0.0648 (7)	0.0843 (8)	0.0560 (6)	0.0065 (6)	0.0285 (5)	0.0192 (5)
S1	0.0518 (6)	0.0429 (5)	0.0431 (5)	−0.0112 (4)	0.0107 (4)	−0.0034 (4)
O1	0.0667 (18)	0.0535 (16)	0.0418 (14)	−0.0241 (14)	0.0165 (12)	−0.0041 (12)
N1	0.0400 (16)	0.0419 (16)	0.0347 (16)	−0.0095 (13)	0.0090 (12)	−0.0012 (12)
N2	0.0476 (17)	0.0416 (16)	0.0320 (15)	−0.0097 (13)	0.0099 (13)	−0.0027 (12)
C1	0.054 (2)	0.049 (2)	0.039 (2)	−0.0039 (18)	0.0112 (17)	−0.0030 (16)
C2	0.053 (2)	0.049 (2)	0.0352 (19)	−0.0072 (18)	0.0104 (17)	0.0039 (16)
C3	0.049 (2)	0.040 (2)	0.0351 (19)	−0.0011 (17)	0.0060 (16)	0.0035 (15)
C4	0.0367 (19)	0.0363 (19)	0.0359 (18)	−0.0024 (15)	0.0017 (14)	0.0057 (14)
C5	0.0395 (19)	0.043 (2)	0.0315 (18)	0.0014 (16)	0.0052 (14)	0.0053 (15)
C6	0.044 (2)	0.062 (2)	0.039 (2)	−0.0111 (18)	0.0075 (16)	0.0024 (18)
C7	0.057 (2)	0.063 (3)	0.033 (2)	−0.001 (2)	0.0095 (17)	0.0000 (17)
C8	0.041 (2)	0.052 (2)	0.042 (2)	0.0070 (17)	0.0135 (16)	0.0141 (17)
C9	0.040 (2)	0.048 (2)	0.052 (2)	−0.0045 (17)	0.0088 (17)	0.0114 (18)
C10	0.045 (2)	0.045 (2)	0.040 (2)	−0.0032 (17)	0.0050 (16)	0.0028 (16)

### Geometric parameters (Å, º)

**Table:**

Cl1—C1	1.780 (4)	C2—C3	1.502 (4)
Cl2—C8	1.741 (3)	C2—H2B	0.9700
S1—C4	1.660 (3)	C2—H2C	0.9700
O1—C3	1.227 (4)	C5—C6	1.376 (5)
N1—C3	1.366 (4)	C5—C10	1.388 (5)
N1—C4	1.389 (4)	C6—C7	1.383 (5)
N1—H1A	0.8600	C6—H6A	0.9300
N2—C4	1.332 (4)	C7—C8	1.370 (5)
N2—C5	1.410 (4)	C7—H7A	0.9300
N2—H2A	0.8600	C8—C9	1.373 (5)
C1—C2	1.506 (4)	C9—C10	1.369 (5)
C1—H1B	0.9700	C9—H9A	0.9300
C1—H1C	0.9700	C10—H10A	0.9300
			
C3—N1—C4	129.6 (3)	N2—C4—N1	114.4 (3)
C3—N1—H1A	115.2	N2—C4—S1	127.3 (3)
C4—N1—H1A	115.2	N1—C4—S1	118.3 (2)
C4—N2—C5	131.6 (3)	C6—C5—C10	118.7 (3)
C4—N2—H2A	114.2	C6—C5—N2	125.5 (3)
C5—N2—H2A	114.2	C10—C5—N2	115.7 (3)
C2—C1—Cl1	111.2 (3)	C5—C6—C7	120.2 (3)
C2—C1—H1B	109.4	C5—C6—H6A	119.9
Cl1—C1—H1B	109.4	C7—C6—H6A	119.9
C2—C1—H1C	109.4	C8—C7—C6	119.7 (3)
Cl1—C1—H1C	109.4	C8—C7—H7A	120.1
H1B—C1—H1C	108.0	C6—C7—H7A	120.1
C3—C2—C1	113.6 (3)	C7—C8—C9	121.0 (3)
C3—C2—H2B	108.8	C7—C8—Cl2	119.1 (3)
C1—C2—H2B	108.8	C9—C8—Cl2	119.9 (3)
C3—C2—H2C	108.8	C10—C9—C8	118.9 (3)
C1—C2—H2C	108.8	C10—C9—H9A	120.6
H2B—C2—H2C	107.7	C8—C9—H9A	120.6
O1—C3—N1	122.7 (3)	C9—C10—C5	121.4 (3)
O1—C3—C2	123.2 (3)	C9—C10—H10A	119.3
N1—C3—C2	114.1 (3)	C5—C10—H10A	119.3
			
Cl1—C1—C2—C3	73.9 (4)	C10—C5—C6—C7	−0.8 (6)
C4—N1—C3—O1	−7.3 (6)	N2—C5—C6—C7	−178.1 (3)
C4—N1—C3—C2	173.8 (3)	C5—C6—C7—C8	0.5 (6)
C1—C2—C3—O1	13.7 (5)	C6—C7—C8—C9	−0.4 (6)
C1—C2—C3—N1	−167.4 (3)	C6—C7—C8—Cl2	179.7 (3)
C5—N2—C4—N1	−177.7 (3)	C7—C8—C9—C10	0.4 (6)
C5—N2—C4—S1	1.6 (6)	Cl2—C8—C9—C10	−179.6 (3)
C3—N1—C4—N2	7.8 (5)	C8—C9—C10—C5	−0.7 (6)
C3—N1—C4—S1	−171.5 (3)	C6—C5—C10—C9	0.9 (5)
C4—N2—C5—C6	−17.2 (6)	N2—C5—C10—C9	178.4 (3)
C4—N2—C5—C10	165.4 (3)		

### Hydrogen-bond geometry (Å, º)

Cg1 is the centroid of the C5–C10 benzene ring.

**Table:**

*D*—H···*A*	*D*—H	H···*A*	*D*···*A*	*D*—H···*A*
N2—H2*A*···O1	0.86	1.92	2.646 (4)	141
C6—H6*A*···S1	0.93	2.55	3.193 (4)	126
N1—H1*A*···S1^i^	0.86	2.52	3.367 (3)	169
C9—H9*A*···O1^ii^	0.93	2.55	3.402 (5)	152
C1—H1*B*···*Cg*1^iii^	0.97	2.92	3.690 (4)	137

Symmetry codes: (i) −*x*+1, −*y*+1, −*z*+1; (ii) −*x*−1, −*y*, −*z*+1; (iii) −*x*, −*y*, −*z*+1.
